# Comparative analysis of two Korean irises (*Iris ruthenica* and *I. uniflora*, Iridaceae) based on plastome sequencing and micromorphology

**DOI:** 10.1038/s41598-022-13528-z

**Published:** 2022-06-08

**Authors:** Bokyung Choi, Inkyu Park, Soonku So, Hyeon-Ho Myeong, Jangseung Ryu, Yu-Eun Ahn, Kyu-Chan Shim, Jun-Ho Song, Tae-Soo Jang

**Affiliations:** 1 Department of Biological Science, College of Bioscience and Biotechnology, Chungnam National University, Daejeon, 34134 Korea; 2 Herbal Medicine Resources Research Center, Korea Institute of Oriental Medicine, Naju, 58245 Korea; 3 Korea National Park Research Institute, 171, Dangu-ro, Wonju-si, Gangwon-do 26441 Korea; 4 Plant Conservation Center, Korea National Park Research Institute, 2 Baengnyeonsa-gil, Seolcheon-Myeon, Muju-gun, Jeollabuk-do 55557 Korea; 5 Department of Agronomy, College of Agriculture and Life Science, Chungnam National University, Daejeon, 34134 Korea

**Keywords:** Evolution, Molecular evolution, Phylogenetics, Speciation

## Abstract

*Iris ruthenica* Ker Gawl. and *I. uniflora* Pall. ex Link, which are rare and endangered species in Korea, possess considerable horticultural and medicinal value among Korean irises. However, discrimination of the species is hindered by extensive morphological similarity. Thus, the aim of the present study was to identify discriminating features by comparing the species’ complete plastid genome (i.e., plastome) sequences and micromorphological features, including leaf margins, stomatal complex distribution (hypostomatic vs. amphistomatic leaves), anther stomata density, and tepal epidermal cell patterns. Plastome comparison revealed slightly divergent regions within intergenic spacer regions, and the most variable sequences, which were distributed in non-coding regions, could be used as molecular markers for the discrimination of *I. ruthenica* and *I. uniflora*. Phylogenetic analysis of the *Iris* species revealed that *I. ruthenica* and *I. uniflora* formed a well-supported clade. The comparison of plastomes and micromorphological features performed in this study provides useful information for elucidating taxonomic, phylogenetic, and evolutionary relationships in Iridaceae. Further studies, including those based on molecular cytogenetic approaches using species specific markers, will offer insights into species delimitation of the two closely related *Iris* species.

## Introduction

Leaf, flower, and pollen micromorphology has been informative for resolving taxonomic problems in angiosperms across various taxonomic levels^1–7^. In particular, leaf epidermal stomata, orbicules, and pollen exine ornamentation characters have been shown to possess systematic values when examined using both light microscopy (LM) and scanning electron microscopy (SEM) and may further be utilized to test phylogenetic hypotheses^8–13^. However, despite an increasing number of the leaf, flower, pollen, and seed micromorphological studies in Iridaceae^14–21^, leaf margins, stomatal occurrence, and orbicular traits have not been considerably challenged for the taxonomic delineation of two closely related species, *Iris ruthenica* and *I. uniflora*. Therefore, understanding their micromorphology may shed light on their taxonomic relationships.

The chloroplast is an essential organelle for photosynthesis, starch and fatty acid biosynthesis, and carbon fixation^22–24^. The length of photosynthetic vascular plant plastomes ranges from 120 to 200 kb and possess a quadripartite structure, with one large single copy (LSC) region, one small single copy (SSC) region, and two inverted repeat (IR) regions. In general, angiosperm plastomes contain 110–130 genes, including approximately 80 protein-coding genes, 30 transfer RNA (tRNA) genes, and four ribosomal RNA (rRNA) genes^22^. These plastomes possess highly conserved structures and gene content and exhibit low variation when compared to nuclear and mitochondrial genomes. However, variable plastome size, gene content, IR expansion or contraction, and structural arrangement have been reported^25,26^. Plastome sequencing can be useful for species classification and identification and high-resolution phylogenetic analysis^27,28^. Facilitated by next-generation sequencing (NGS), plastome data are increasingly utilized for the investigation of phylogenetic relationships and for the development of DNA barcode markers for low-taxonomic-level identification and the discrimination of controversial taxa^29–31^. However, even though complete plastome data are available for a variety of Korean irises^32,33^, neither the detailed comparative analysis of *Iris* plastomes nor the combination of such analysis with micromorphological analysis has been conducted in the genus *Iris* as it has been in other taxonomic groups^34–36^.

**Figure 1:**
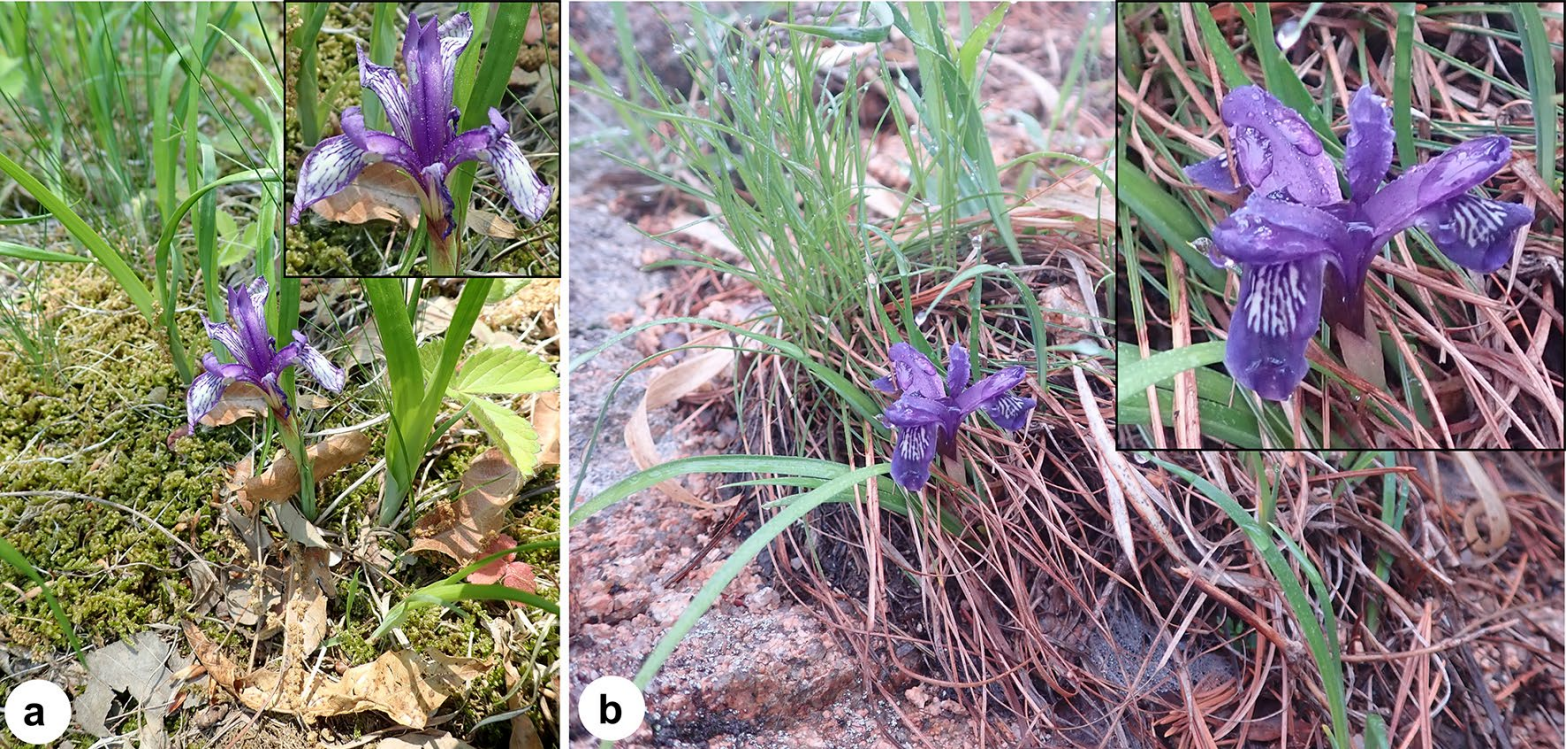
Morphology and habit of *Iris* species from Korea. **(a)** *I. ruthenica*; **(b)** *I. uniflora*. Insets in (**a**) and (**b**) show the enlarged image of the flowers of the investigated species. All photographs were obtained by Tae-Soo Jang.

The genus *Iris* L. contains approximately 300 perennial species, which are distributed in temperate regions across the Northern Hemisphere, as well as a large number of infraspecific taxa^15,37–39^. Based on recent molecular phylogenetic analyses, the genus has been divided into six subgenera^40–45^, including *Iris* L. subg. *Limniris* (Tausch) Spach ser. *Ruthenicae* Diels, which contains two species, *I. ruthenica* Ker Gawl. and *I. uniflora* Pall. ex Link^37,45,46^. Interestingly, both *I. ruthenica* and *I. uniflora* are rare or endangered in Korea (Fig. 1), owing to their similar pharmacological effect as the herbal medicines or because of their popularity as ornamentals^47,48^. However, even though the infrageneric classification of *Iris* based on chloroplast DNA sequence data remains somewhat controversial^33,38,45^, the monophyly of ser. *Ruthenicae* is strongly supported by molecular evidence (whole plastome sequences^33^), chromosome number^46^, and external morphology^37^, and the series can be easily distinguished from other series in the genus^37^. *Iris ruthenica* is arguably most closely related to *I. uniflora* due to their similar morphology^37,47^. Regarding the cytological features, the two species have consistent chromosome numbers (2*n* = 42), and similar genome sizes (2.42 pg/1C in *I. ruthenica*; 2.46 pg/1C in *I. uniflora*)^46^. However, despite of the economic significance of the two species, genomic resources for the ser. *Ruthenicae* are still limited. Interestingly, some taxonomists considered *I. uniflora* as a synonym of *I. ruthenica* due to their high morphological similarity^37,49^, and such an example can be found in other taxonomic groups in the genus *Iris*^50^. Thus, comparison of the genomic data and micromorphology of the two taxa is needed as indicated in recent studies^41,44,46,51,52^.

Accordingly, the aims of the present study were to (1) compare the macro/microscopic features of *I. ruthenica* and *I. uniflora*, (2) characterize and compare the complete de novo-assembled plastomes for both species, and (3) investigate the evolutionary relationships within *Iris* through phylogenetic analysis^33^.

## Results

### Leaf, flower, pollen, and orbicule morphological characters

**Figure 2:**
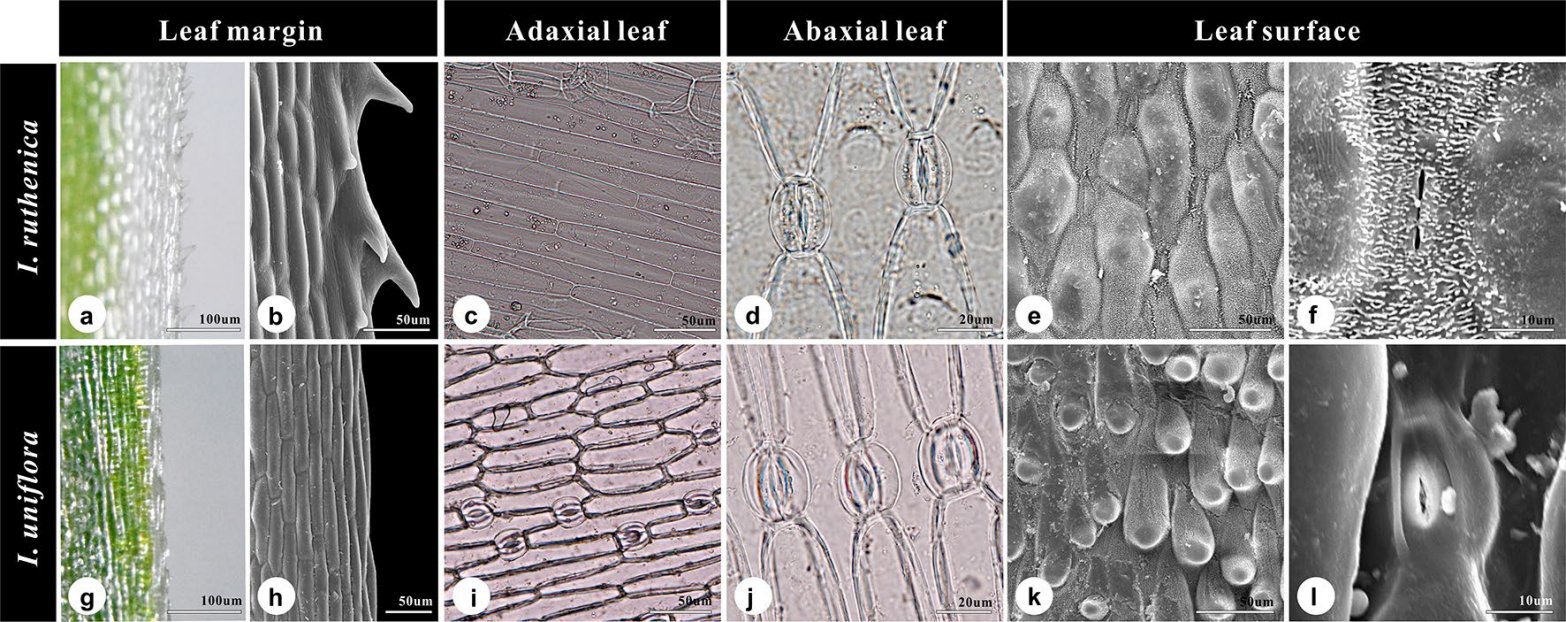
Stereo, light, and scanning micrographs of leaves of *Iris ruthenica* (**a**–**f**) and *I. uniflora* (**g**–**l**). Serrate (**a**,**b**) and entire (**g**,**h**) leaf margin. Hypostomatic (stomata is absent on adaxial surface but present on the abaxial surface of leaf) (**c**,**d**) and amphistomatic (stomata present on both adaxial and abaxial leaf epidermis) leaves (**i**,**j**) with anomocytic stomata. (**e**,**f**, **k**,**l**) Detailed leaf epidermal cell shape and sunken stomata on the leaf surface.

**Figure 3:**
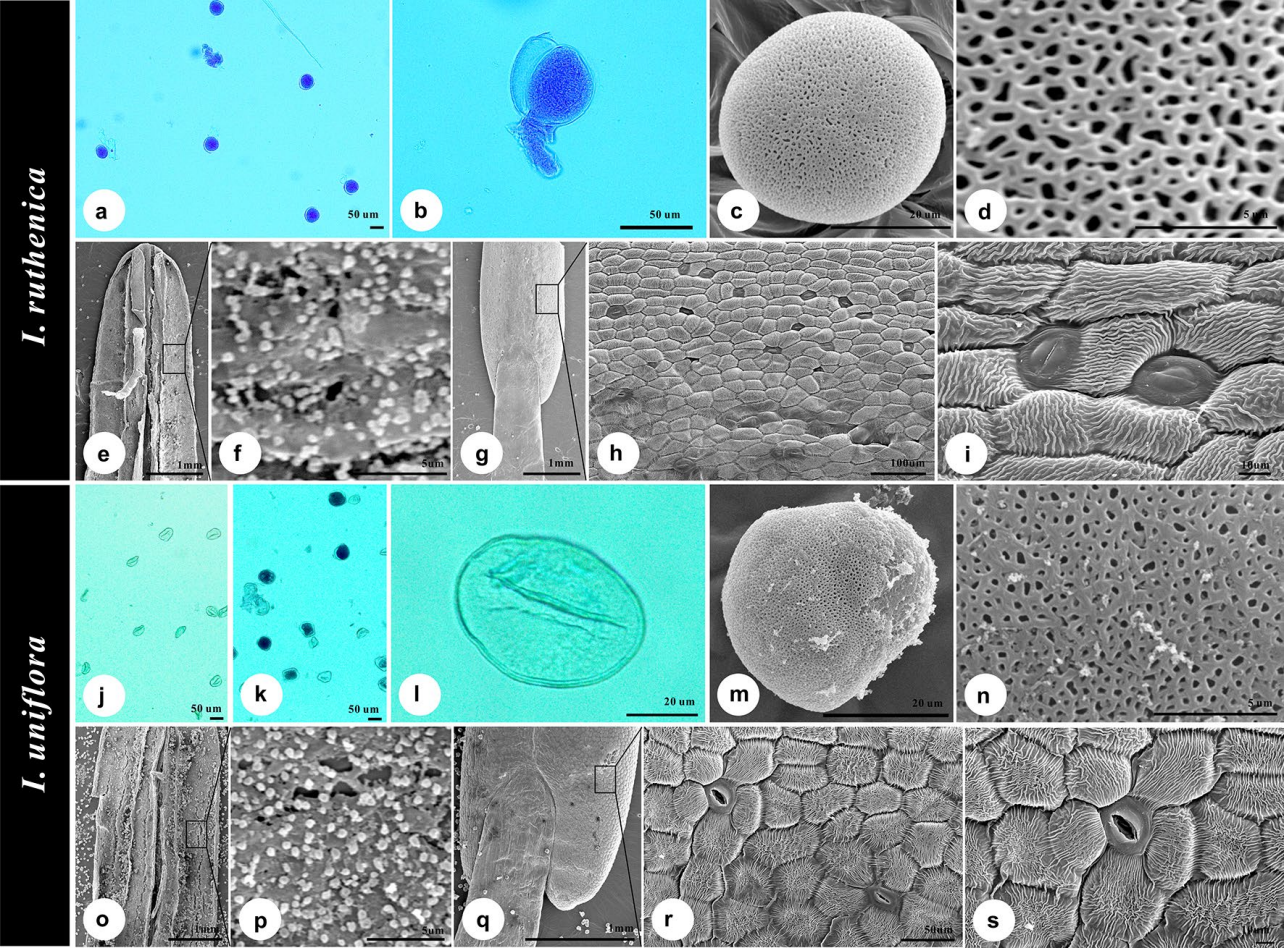
Stamen, pollen, and orbicule micromorphology of *Iris ruthenica* (**a**–**i**) and *I. uniflora* (**j**–**s**). (**a**–**d**, **j**–**n**) Stained fertile (**a**,**b**) or sterile (**j**–**l**) pollen grains and their detailed sexine ornamentation; (**e**,**f**, **o**,**p**) dissected anther (inner part; **e**, **o**) and the occurrence of orbicules (**f**, **p**), respectively; (**g**–**i**, **q**–**s**) outer epidermis of anthers (**g**, **q**) and the occurrence of stomatal complex (**h**,**i**,**r**,**s**), respectively. All photographs were obtained by Bokyung Choi.

The two species possessed similar floral traits (e.g., tepal color, shape, and size; Fig. 1 and Supplementary Fig. S1) and similar leaf epidermis, epidermal cell, and anticlinal wall shapes (Fig. 2). The leaf epidermis of both species consisted of long tabular cells, typically with elongated pavement cells (Fig. 2c,i), with undulate anticlinal walls (Fig. 2e,k) that were covered by either prominent (*I. ruthenica*; Fig. 2f) or weak (*I. uniflora*; Fig. 2l) epicuticular wax. However, the leaf margin of *I. uniflora* was clearly entire, whereas that of *I. ruthenica* was spiny with sharp stiff points (Fig. 2), and the leaves of *I. ruthenica* were hypostomatic (stomata are absent or extremely rare on the adaxial leaf surface while they are present on the abaxial leaf surface; Fig. 2c,d), whereas those of *I. uniflora* were amphistomatic (stomata are present on both adaxial and abaxial leaf epidermis; Fig. 2i–j). In addition, the width of guard cells ranged from 24.63–28.19 μm on the abaxial surfaces of *I. ruthenica* leaves and from 26.53–28.14 μm and 27.02–27.77 μm on the adaxial and abaxial surfaces of *I. uniflora* leaves, respectively (Tables 1, 2). SEM analysis revealed that stomata of both species were clearly sunken (Fig. 2f,l), and that they were all anomocytic lacking subsidiary cells, surrounded by four epidermal cells (Fig. 2d,i,j). Tepal epidermal cells had unicellular covered by a striated cuticle and slightly sunk stomata (Supplementary Fig. S1). Furthermore, the outer anther epidermal cells were polygonal in shape and covered by a striated cuticle in both species, *I. ruthenica* and *I. uniflora* (Fig. 3g,h,q,r). Both species possessed anthers with anomocytic stomata in the middle section, despite differences in stomatal density (Fig. 3h,r). The guard cell surfaces of *I. uniflora* were weakly wrinkled (Fig. 3i), whereas those of *I. uniflora* were smooth (Fig. 3s).

**Table 1:** Plant material information for micromorphological and molecular analyses.

Species	Accession No.	Voucher information; collector	Methods applied for analysis
*I. ruthenica*	BKC939	Chungnam, Korea; BC, T-SJ	CP, M
	JCKC190507	Is. Je-Ju, Korea; BC, T-SJ, SS	M
	JC532	Dae-Gu, Korea; BC, T-SJ	M
*I. uniflora*	JCK2019-77	Mt. Sorak, Kangwon, Korea; BC, T-SJ	CP, M
	JCK2019-78	Mt. Sorak, Kangwon, Korea; BC, T-SJ	M
	SA519	Mt. Sorak, Kangwon, Korea; BC, T-SJ, JR	M

Collectors: BC, Bokyung Choi; JR, Jangseung Ryu; SS, Soonku So; T-SJ, Tae-Soo Jang. Methods applied for analysis: CP, chloroplast genome sequence analysis; M, microscopic analysis.

**Table 2:** Overview of leaf and pollen morphological characters of *Iris ruthenica* and *I. uniflora* examined using light microscope.

Species	Leaf margin	Leaf epidermal stomata			Size and ratio of fertile pollen grains			Size and ratio of sterile pollen grains		
		O	Adaxial	Abaxial	P	E	P/E	P	E	P/E
***I. ruthenica***										
JCKC190507	S	H	–	25.22 ± 2.57	53.71 ± 2.36	53.55 ± 2.63	1.00 ± 0.05	–	–	–
JC532	S	H	–	28.19 ± 1.54	52.13 ± 2.87	51.76 ± 3.20	1.00 ± 0.04	–	–	–
BKC939	S	H	–	24.63 ± 2.05	49.57 ± 4.24	49.32 ± 1.67	1.00 ± 0.07	–	–	–
***I. uniflora***										
JCK2019-77	E	A	28.14 ± 1.34	27.77 ± 2.48	51.99 ± 3.75	49.90 ± 2.51	1.04 ± 0.05	52.54 ± 7.49	36.27 ± 6.33	1.49 ± 0.35
JCK2019-78	E	A	26.92 ± 1.66	27.02 ± 2.68	56.31 ± 4.10	50.62 ± 4.45	1.12 ± 0.12	47.61 ± 5.37	40.08 ± 4.99	1.19 ± 0.12
SA519	E	A	26.53 ± 2.40	27.19 ± 1.96	48.95 ± 3.46	51.66 ± 3.49	0.94 ± 0.03	47.54 ± 2.28	39.18 ± 4.66	1.22 ± 0.14

Leaf margin: S, serrate; E, entire. Leaf epidermal stomata occurrence (O): A, amphistomatic; H, hypostomatic or absent or extremely rare. Size and ratio of pollen grains: P, polar diameter; E, equatorial diameter; P/E, polar diameter/equatorial diameter. All size measurements are in µm (mean ± standard deviation).

The pollen grains of both species were monads of 47.54–56.31 μm in polar length and 39.18–53.55 μm in equatorial diameter (Table 2). While both species yielded fertile pollen grains (Fig. 3a,k), sterile pollen grains were only found extremely rarely or absent in *I. ruthenica* (Fig. 3a–c), and, in *Iris uniflora*, sterile pollen grains were frequently encountered (Fig. 3j–l), which differed from the fertile pollen grains of both species in regard to equatorial width (Table 2), shape (subprolate to prolate, P/E = 1.19–1.49 vs. oblate-spheroidal to prolate-spheroidal, P/E = 0.94–1.12; Table 2), and exine ornamentation (irregularly microreticulate exine ornamentation vs. monosulcate with microreticulate ornamentation; Fig. 3c–d,l–n). The orbicules of both species were entirely fused with the inner locule anther wall, particularly at the tapetal membrane (Fig. 3f, p), and possessed almost identical morphology (i.e., density, size, shape, and surface details).

### Plastome sequencing

Illumina MiSeq yielded 5.2 and 6.0 Gb raw paired-end (2 × 300 bp) reads for *I. ruthenica* and *I. uniflora*, respectively, and 2.8 and 4.5 Gb trimmed reads, thereby providing coverage of approximately 739.3 × and 1064.6 × , respectively (Supplementary Tables S6 and S7).

### Plastome characteristics

**Figure 4:**
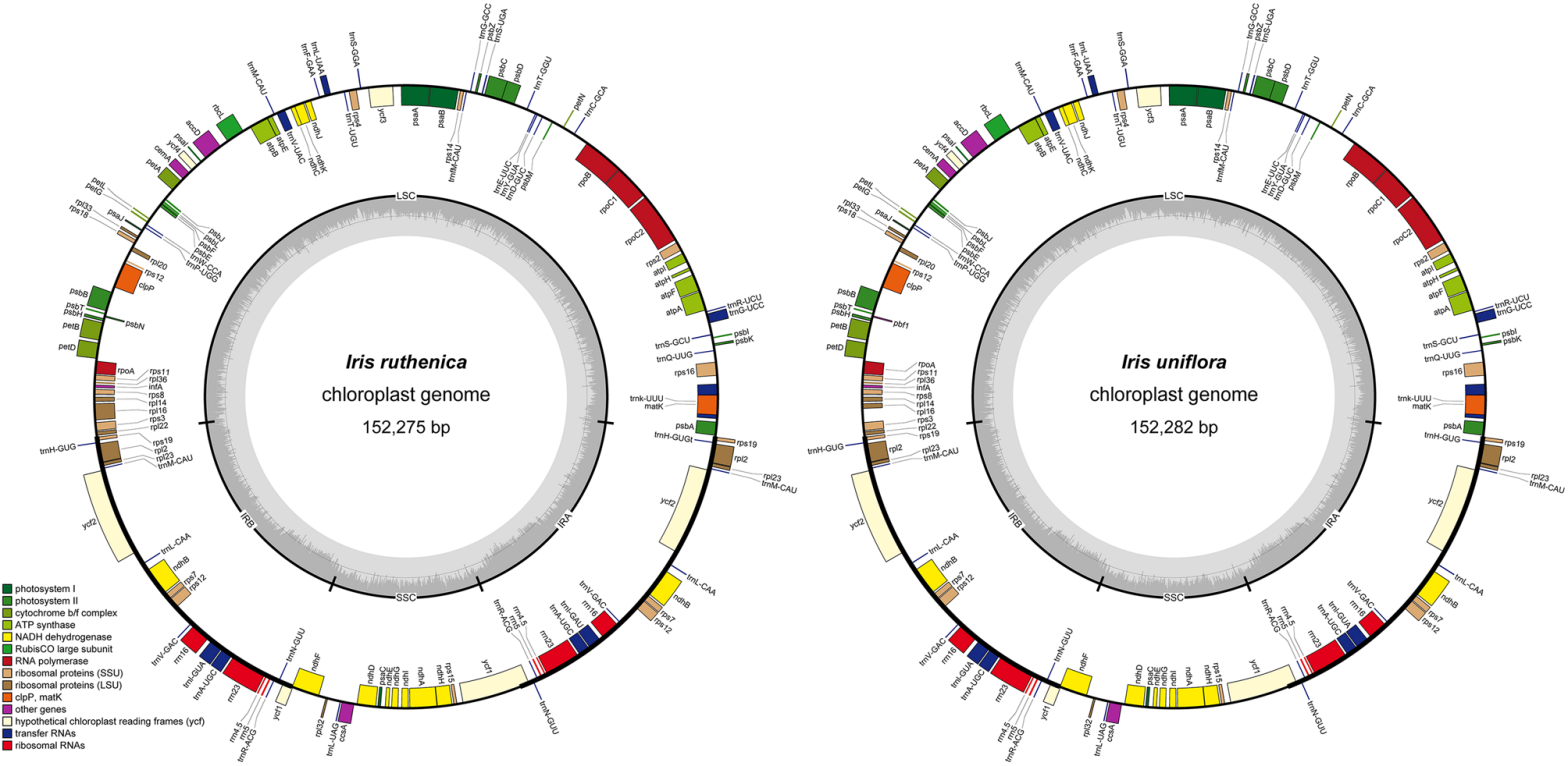
Circular gene maps of *Iris ruthenica* and *I. uniflora* plastomes*.* Genes drawn inside the circle are transcribed clockwise, and those outside the circle are transcribed counterclockwise. The darker gray in the inner circle represents GC content.

Both plastomes exhibited the quadripartite structure typical of angiosperm taxa (Fig. 4), with total lengths of 152,275 and 152,282 bp, LSC region lengths of 82,301 and 82,307 bp, and SSC region lengths of 18,134 and 18,135 bp in *I. ruthenica* and *I. uniflora*, respectively, as well as an IR region of 25,920 bp in length in both species (Table 3, Supplementary Figs. S2–4). The plastome junction regions were validated through the generation of high-quality plastome sequences. The overall GC content of the two plastomes was 38.1%, with greater GC content in the IR regions of *I. ruthenica* and *I. uniflora* (43.3 and 43.2%, respectively) than in the LSC regions (36.3 and 36.3%) and SSC regions (32.2 and 32.1%). Both plastomes contained 115 genes (80 protein-coding, 4 rRNA, and 31 tRNA genes; Table 3), 18 intron-containing genes (16 with one intron and two with three introns), and duplicate genes (*ndhB*, *trnI-GAU*, and *trnA-UGC*) in the IR regions (Supplementary Table S9). Analysis of codon usage and anticodon recognition patterns indicated that the plastomes of *I. ruthenica* and *I. uniflora* contained 26,634 and 26,641 codons, respectively, and that leucine, isoleucine, and serine were the most abundant (Supplementary Fig. S2). Relative synonymous codon usage (RSCU) analysis indicated synonymous codon bias, with a high proportion of A or T in the third position. Most RSCU values indicated a similar pattern. RSCU values for arginine were usually high.

**Table 3:** Summary of the major characteristics of the chloroplast genomes of *I. ruthenica* and *I. uniflora*.

Species	*I. ruthenica*	*I. uniflora*
Total cp genome size (bp)	152,275	152,282
Large single copy (LSC) region (bp)	82,301	82,307
Inverted repeat (IR) region (bp)	25,920	25,920
Small single copy (SSC) region (bp)	18,134	18,135
Total number of genes (unique)	114	114
Protein-coding gene (unique)	80	80
rRNA (unique)	4	4
tRNA (unique)	31	31
GC content (%)	38.1%	38.1%
LSC (%)	36.3%	36.3%
IR (%)	43.3%	43.2%
SSC (%)	32.2%	32.1%

### Plastome comparison

**Figure 5:**
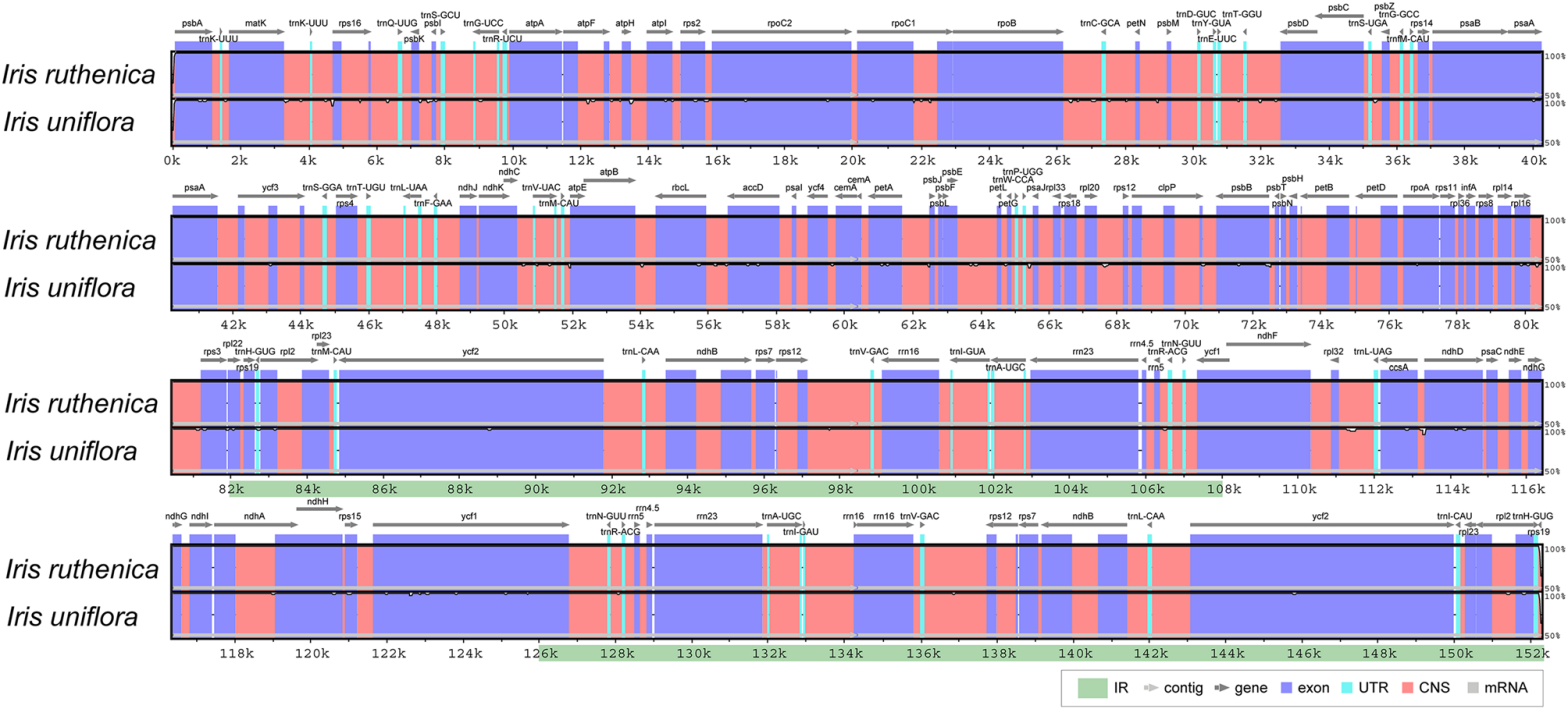
Comparison of *Iris ruthenica* and *I. uniflora* plastomes using mVISTA. Complete plastomes of *I. ruthenica* and *I. uniflora* were compared to that of *I. ruthenica*. Blue block: conserved genes; sky-blue block: transfer RNA (tRNA) and ribosomal RNA (rRNA) genes; red block: conserved non-coding sequences (CNS). Regions with sequence variation between *I. ruthenica* and *I. uniflora* are denoted in white. Horizontal axis indicates coordinates within plastomes. Vertical scale represents percent identity, ranging from 50–100%.

**Figure 6:**
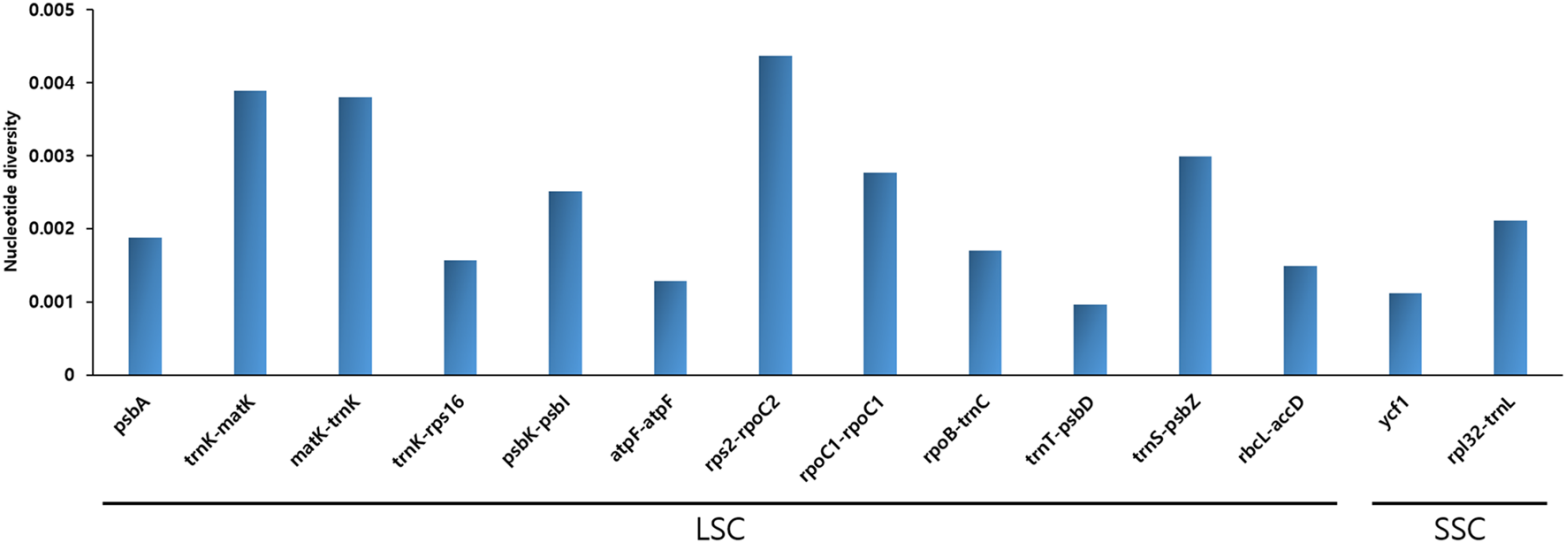
Comparison of nucleotide diversity (Pi) values among *Iris ruthenica* and *I. uniflora* species and parsimony haplotype analyses using *psbA*, *matK-trnK*, and *psbK-psbl*.

Plastome alignment revealed slight genomic variation, with intergenic regions being the most divergent. However, the plastomes generally formed a well-conserved collinear block (Supplementary Fig. S3), with a highly conserved structure. Nucleotide diversity analysis identified 14 regions with weak variation (Figs. 5, 6). The genes *psbA* and *ycf1* yielded Pi values of 0.00188 and 0.00112, respectively. Most of the divergent regions were located in the LSC region (Pi = 0.00243). In particular, the *trnK-matK*, *matK-trnK*, and *trnK-rps16* regions have shown consecutive nucleotide variations in the LSC region. The analysis of IR boundaries revealed that *rpl22* was located in the LSC region of both species. The *ycf1* and *ndhA* genes were located in the junction of SSC/IRb region. The *rps19* gene, which was duplicated in the IR regions, was generally well-conserved. The *ycf1* gene was located at the IRa/SSC and SSC/IRb junctions (Supplementary Fig. S4).

### Phylogenic relationships among *Iris* species

**Figure 7:**
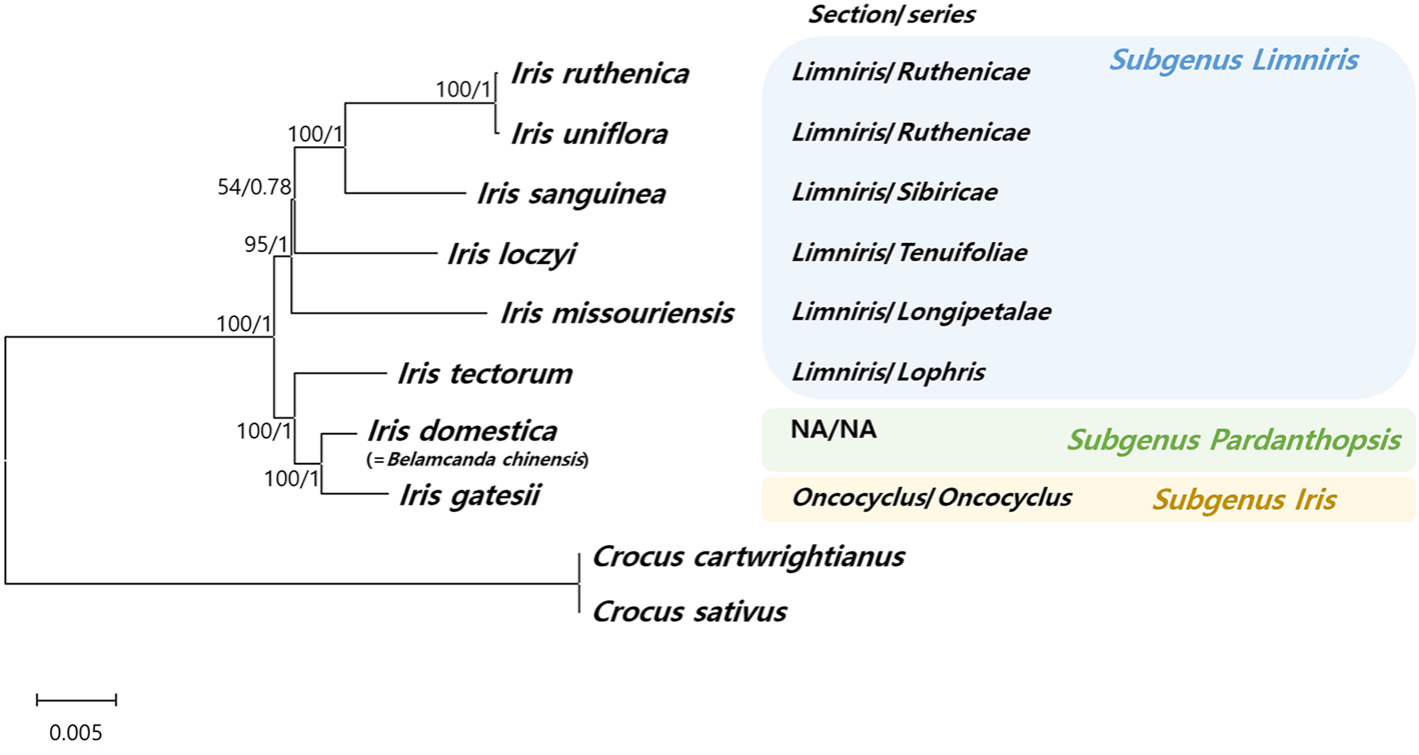
Phylogenetic tree of *Iris* species constructed using maximum likelihood bootstrap analysis and Bayesian posterior probability. Maximum likelihood topology is shown with bootstrap support values and Bayesian posterior probabilities given at each node.

The ML and BI topologies were highly congruent for the whole plastome and CDS datasets, and all but one lineage was strongly supported (ML > 95%, BI = 1.0). More specifically, the topologies were clearly divided into three major clades, which corresponded to the subgenera *Limniris*, *Pardanthopsis*, and *Iris* (Fig. 7). Most of the *Iris* species included in the present study were assigned to section *Limniris*, which is consistent with the Angiosperm Phylogeny Group (APG) IV classification system, except for *I. tectorum*^53^. Furthermore, a clade containing *I. domestica* and *I. gatesii* was clustered as a sister group to *I. tectorum*; *I. ruthenica* and *I. uniflora* formed a monophyletic clade (Fig. 7).

## Discussion

### Micromorphology

*Iris ruthenica* and *I. uniflora* are sister taxa within ser. *Ruthenicae* and, as such, share a variety of morphological characters, including creeping rhizomes, leaf shape and size, flower color, and fruit shape^37^. Indeed, due to their morphological similarity, the taxonomic status of the species has been controversial. For example, Zhao et al.^37^ suggested that *I. uniflora* be considered a subgroup of *I. ruthenica*, and Zheng et al.^54^ argued that morphological differences between the species are the result of environmental conditions (i.e., different habitats). However, in the present study, *I. ruthenica* and *I. uniflora* could be differentiated using a variety of micromorphological features, including stomatal complex distribution (hypostomatic leaves vs. amphistomatic leaves), slight differences in the protruded conical cells of leaf surfaces, pollen grain fertility (all fertile vs. both fertile and sterile), and anther stomata density.

The micromorphological analysis performed in the present study revealed that *I. ruthenica* and *I. uniflora* possess leaves with an irregular shape and sinuate anticlinal striation, as previously reported^14,20,55^. Because both leaf surface micromorphology and stomata have shown considerable variation in papillae, they are not taxonomically significant, as suggested by Wu and Cutler^14^ and other monocot species^56,57^. The papillae that cover stomata likely contribute to defense against unfavorable environmental conditions^58^ or attack by pathogens^59^. Papillae can also play an important role in light reflection, by preventing overheating^60^, and, as such, may be more correlated with environmental conditions than taxonomic status. Regardless of stomatal distribution, both species possessed anomocytic stomata (Table 2), which is likely the ancestral (plesiomorphic) condition among monocots^61^. The guard cell and genome sizes of the two *Iris* species included in the present study were not significantly distinct (Table 2; Choi et al.^46^), although it is well known that stomata size is positively correlated with genome size and that ploidy level changes in plants^62–64^.

With the exception of clear differences in pollen viability, the morphology (e.g., shape, size, exine pattern, and orbicule presence) of pollen in *I. ruthenica* and *I. uniflora* were not significantly different (Fig. 3). As *I. uniflora* was only found under low-temperature stress conditions, whereas *I. ruthenica* was widely distributed^46–48^, the occurrence of sterile pollen grains in *I. uniflora* might be affected by meiotic abnormalities with genetic constitution as reported in other cold regions^65,66^. Orbicules, also known as Ubisch bodies, are small sporopollenin particles that can be produced on the inner locule walls of anthers^67^ and that are, here, reported in *I. ruthenica* and *I. uniflora* for the first time. Even though orbicule morphology has been widely used to elucidate systematic and evolutionary relationships^12,68,69^, the occurrence of orbicules has, until now, only been reported in *I. pallida* Lam.^70^. Thus, further investigation of orbicule occurrence in the Iridaceae might yield a significant phylogenetic trait, as reported in other plant groups^67–72^. However, as with other morphological features of *Iris* taxa, such as tepal structure^17^, pollen morphology^15,16,21,73–75^, and seed microstructure^18^, the micromorphological features of either vegetative or reproductive organs are largely insufficient for reconstructing the taxonomic relationship of *I. ruthenica* and *I. uniflora*.

### Plastome characterization and variation

The structures of the *I. ruthenica* and *I. uniflora* plastomes were similar to those of plastomes from other members of the Iridaceae^32,33^, with the typical quadripartite structure and LSC and SSC regions separated by IR regions. The plastomes of the *Iris* species contained 114 unique genes, and their gene order, GC content, genomic structure, and overall length (152,275 and 152,282 bp) were within the ranges previously described for *Iris* plastomes^76^.

The mVISTA results indicated that the *Iris* plastomes contained little variation and that genic regions were more conserved than IGS regions, which is consistent with angiosperm plastomes in general^77–79^. More specifically, the *psbA*, *trnK-matK*, *matK-trnK,* and *trnK-rps16* regions were hotspots for genetic variation (Fig. 5), which indicated underlying evolution^80–83^ and value as molecular markers^84,85^. In terms of nucleotide diversity (Pi), most of the divergent regions were non-coding, which is consistent with previous reports^86–88^. Other plastomes were highly variable for the non-coding regions at *psbA*, *trnK-matK*, *matK–trnK*, and *trnK-rps16* in the present study (Fig. 6). mVISTA and Pi analysis showed difference between *I. ruthenica* and *I. uniflora* at the plastome level. Furthermore, these regions will play an important role in the discrimination of *I. ruthenica* and *I. uniflora*, as well as other species within the Iridaceae.

IR contraction and expansion causes variation in the size of angiosperm plastomes^89^. Previous studies have reported extremely short IRs or the loss of IR regions and genes^90,91^. Compared to *I. ruthenica*, *I. uniflora* had a highly conserved IR length and gene positions. However, the *rps19* genes of *I. ruthenica* and *I. uniflora* were located in the IRa region, and *ycf1* was located in the IRa/SSC, region which overlapped with *ndhF.* Thus, the *Iris* plastome possessed an extended IR, as reported previously^32,33^.

### Phylogenetic relationships in the Iridaceae

Plastid genome (i.e., plastome) sequences are valuable genomic resources for estimating phylogenetic relationships, particularly among closely related species and unresolved taxa^4,27,31^. The systematics of Korean irises have been widely discussed, and several molecular studies based on single molecular markers (e.g., *psbA-trnH*, *trnL-F*) and plastome structure have been performed in the past^33,92^. The topologies of phylogenetic trees based on whole plastome sequences in the present study are similar to those reported previously (Fig. 7, Supplementary Figs. S5–S7)^33,41,45,52,92^. In agreement with earlier results, *I. ruthenica* and *I. uniflora* were nested deeply within section *Limniris* ser. *Ruthenicae*. However, in the present study, the monophyly of section *Limniris* was compromised by the inclusion of *I. tectorum* (ser. *Lophris*), which was deeply nested with subgenera *Pardanthopsis* and *Iris* in both the ML and BI trees (Fig. 7). Due to insufficient taxonomic sampling, the results of the present study are not suitable for the discussion of inter-subgeneric relationships within *Iris s.l.* ^44^. Nevertheless, the present study provides important genetic resources for further studies within the genus, as well as micromorphological comparisons between two closely related species in a taxonomic context.

The results of the present study confirm that *Iris* section *Limniris* ser. *Ruthenicae* (formerly section *Ioniris* sensu Zhao et al.^37^) contains the two species, *I. ruthenica* and *I. uniflora*. Morphologically, *I. uniflora* is distinguished from *I. ruthenica* because of the presence of narrow leaves^54^, but the flowers of the species are difficult to distinguish. In Korea, *I. ruthenica* is widespread, while *I. uniflora* is restricted to alpine areas^46–48^. Based on the comparison of morphological and chloroplast genome data of the two species, it is doubtful whether *I. ruthenica* and *I. uniflora* can be recognized as independent species-level taxa, as suggested by Zheng et al.^54^. Thus, further studies on molecular and morphological analyses of the two species at population level are required to clarify the taxonomic status of the two taxa.

## Conclusions

The present study provides detailed insights into the leaf and flower micromorphologies and plastome structures of the two closely related species *I. ruthenica* and *I. uniflora*. Micromorphological features, including leaf margins, stomatal complex distribution (hypostomatic vs*.* amphistomatic leaves), anther stomata density, and floral epidermis cell patterns, are somehow useful for distinguishing the taxa, despite that the drastic influences of environmental variation, especially climate factors (e.g., temperature and light intensity), may also contribute to the morphological variations. The plastome sequences of the two related species possessed similar genome lengths, gene numbers, and gene orientations. Most of the variable sequences, which were found in non-coding regions, could be used as molecular markers for the differentiation of *I. ruthenica* and *I. uniflora*, as well as other *Iris* taxa. Given the economic and ecological importance of Korean *Iris* species, the molecular phylogenetic studies can now prompt a search for diagnostic characters, such as general morphological and micromorphological traits, a necessary prerequisite for any systematic and taxonomic context. Further integrative analyses of plastome sequences and morphological data of the two species at population level as well as employing molecular cytogenetic approaches using species-specific satellite DNA as probes may also offer insights into the species delimitation of the two closely related *Iris* species.

## Materials and methods

### Taxon sampling

All plant materials were collected from natural populations in Korea (Table 1). Assoc.-Prof. Dr. Tae-Soo Jang and Dr. Soonku So formally identified all the samples. Considering the protection of Korean endangered plant resources, we only collected a small number of plant specimens with the approval and permission of the local authorities (collection permit nos. 2019–13 [JC532], 2019–14 [BKC939], 2019–20 [JCKC190507]; Table 1). To evaluate the consistency of morphological and micromorphological characters, living specimens were collected from multiple populations (three *I. ruthenica* specimens from three populations and three *I. uniflora* specimens from one population), and cultivated at Chungnam National University (Table 1). Meanwhile, for plastome sequencing, representative fresh leaves were collected from *I. ruthenica* and *I. uniflora* specimens (accession numbers: BKC939 and JCK2019-77, respectively; Table 1). Specific locality information including GPS coordinates, latitude, and longitude cannot be provided due to the endangered/rare status of the species in Korea. All voucher specimens were deposited in the Chungnam National University Herbarium (CNUK).

### Micromorphological analysis

Fresh leaf and flower materials from all four *Iris* populations were preserved using a formalin-acetic acid-alcohol solution and dehydrated by soaking in an acetone series (50, 70, and 90%) for 30 min and absolute acetone for 1 h. The dehydrated materials were then immersed in carbon dioxide for critical point drying (EMCPD300, Leica Microsystems, Germany), coated using an ion-sputtering device (E-1010, Hitachi, Japan), and analyzed using scanning electron microscopy (SEM; S3000N, Hitachi, Japan), with an accelerating voltage of 20 kV and working distance of 9–15 mm, following Choi et al.^5^, as well as light microscopy (BX53F, Olympus, Japan), which was used to analyze the structure of leaf epidermal pavement cells and stomatal complexes, following Kim et al.^7^. At least 20 guard cells were examined on both adaxial and abaxial leaf surfaces from each sample as described by Choi et al.^5^. To measure the pollen viability of each species, 10 randomly selected anthers from each individual plant were placed in an aniline blue dye solution to distinguish fertile pollen grains as described by Jang et al.^93^. At least 20 sterile and fertile pollen grains from each sample were randomly selected for size measurements. Both leaf and pollen micromorphological characters were measured using MicroMeasure ver. 3.3 program following Jang et al.^94^. For SEM, imaging was performed for both the abaxial and adaxial leaf surfaces, as well as for both the inner and outer surfaces of the outer tepals, stamens, and pollen grains. Orbicules, which are cellular structures of sporopollenin particles produced by the secretory tapetum, were investigated in this study for the first time using SEM in the genus *Iris*.

### Plastome sequencing and assembly

Total genomic DNA was extracted from freshly collected samples using the modified CTAB method^95^, and Illumina short-insert paired-end sequencing libraries (TruSeq DNA Nano kit, Illumina, San Diego, CA, USA) were constructed and sequenced using the Illumina MiSeq platform. For de novo plastome assembly, sequencing reads were trimmed and filtered using FastQC v0.11.7^96^, and the resulting trimmed paired-end reads (Phred score ≥ 20) were assembled using Velvet 1.2.10^97^, with kmer values of 71, 91 101, and 111 to form large contigs. The Velvet contigs were then assembled into complete plastomes using the de novo assembly option in Geneious prime (https://www.geneious.com) and ordered using reference plastome sequences from *I. gatesii* (NC_024936), *I. sanguinea* (NC_029227), and *I. missouriensis* (NC_042827). Finally, the LSC/IR, IR/SSC, SSC/IR, and IR/LSC regions of the complete plastomes were validated using PCR-based sequencing. Primer information and sequence alignment results are listed in Supplementary Tables S1 and S2.

### Plastome annotation and repeat sequence analysis

The *I. ruthenica* and *I. uniflora* plastomes were annotated using GeSeq^98^. Protein-coding sequences were manually curated and confirmed using Artemis^99^ and then checked against the NCBI protein database. tRNA genes were confirmed using tRNAscan-SE 1.21^100^, and IR region sequences were confirmed using IR finder and RepEx^101^. Finally, circular maps of the *I. ruthenica* and *I. uniflora* plastomes were generated using OGDRAW^102^.

For plastome comparison, GC content and relative synonymous codon usage (RSCU) were calculated using MEGA6^103^. The two plastomes were also compared using mVISTA in Shuffle-LAGAN mode, with *I. ruthenica* plastome as a reference, and nucleotide variation (Pi) among the plastomes, excluding regions of < 200 bp, was calculated using DnaSP version 6.1^104^. Each plastome was divided into genes, introns, and intergenic regions.

### Plastome phylogenetic analysis

The plastome sequences of eight taxa, including six other taxa from the Iridaceae and the outgroup taxa *Crocus cartwrightianus* (NC_041459) and *C. sativus* (NC_041459), were obtained from NCBI GenBank (Supplementary Table S3), and two matrices, one of whole plastome sequences and another of 78 conserved protein-coding sequences (CDS), which excluded duplicate genes in the IR region, were generated using MAFFT ver. 7^105^; all ten plastome sequences were then manually adjusted using Bioedit^106^. For CDS analysis, the aligned CDS were extracted and concatenated using Geneious (https://www.geneious.com) and filtered to remove ambiguously aligned regions using GBLOCKS ver. 0.91b^107^. The best-fitting model for nucleotide substitution was determined using the Akaike Information Criterion (AIC) in JModeltest V2.1.10^108^ (Supplementary Table S4). Maximum likelihood (ML) analysis was performed using RaxML v8.0.5^109^, with 1000 bootstrap replicates and the GTR + I + G model, and Bayesian Inference (BI) analysis was performed using MrBayes 3.2.2^110^, with two independent runs and four simultaneous Markov Chain Monte Carlo runs of 5,000,000 generations each. The resulting trees were sampled every 100,000 generations, with the first 25% discarded as burn-in, and the 50% majority-rule consensus tree was visualized using Figtree V.1.4.2^111^, with posterior probability (PP) values estimated from trees sampled after the burn-in fraction was discarded.

### Ethics approval

The experimental research and field studies on plants, including the collection of plant material, complied with relevant institutional, national, and international guidelines and legislation. The appropriate permissions and/or licenses for collection of plant were obtained for the study.

## Supplementary Information

Supplementary Information.
