# Supplementary material for: Comparative analysis of two Korean irises (Iris ruthenica and I. uniflora, Iridaceae) based on plastome sequencing and micromorphology
Source: Sci Rep. 2022 Jun 8;12:9424. doi: 10.1038/s41598-022-13528-z (PMC9177672; doi:10.1038/s41598-022-13528-z)
Supplement: Supplementary file 1 — Supplementary Information. [file 41598_2022_13528_MOESM1_ESM.doc]

**SUPPLEMENTARY INFORMATION**

**Comparative analysis of two Korean irises (*Iris ruthenica* and *I. uniflora*, Iridaceae) based on plastome sequencing and micromorphology**

**Bokyung Choi^1†^, Inkyu Park^2†^, Soonku So^3^, Hyeon-Ho Myeong^4^, Jangseung Ryu^1^, Yu-Eun Ahn^1^, Kyu-Chan Shim^5^, Jun-Ho Song^2^, Tae-Soo Jang^1*^**

^1^Department of Biological Science, College of Bioscience and Biotechnology, Chungnam National University, Daejeon 34134, Korea.

^2^Herbal Medicine Resources Research Center, Korea Institute of Oriental Medicine, Naju 58245, Korea.

^3^National Park Research Institute, 171, Dangu-ro, Wonju-si, Gangwon-do Korea 26441.

^4^Department of Agronomy, College of Agriculture and Life Science, Chungnam National University, Daejeon 34134, Korea.

^†^These authors (Bokyung Choi and Inkyu Park) have contributed equally to this work.

***** Correspondence: Tae-Soo Jang (jangts@cnu.ac.kr)

**Supplementary Table S1.** Primers used in this study for chloroplast junction validation.

| Primer name | Primer sequence (5`>3`) | Position |
| --- | --- | --- |
| LI_F | GCACTTTGAAAACAGACATAGGC | LSC_IRa |
| LI_R | GCTGTAGCGAAGCTGATTGC |
| IS_F | GCGCCTCTGCATCTAGCATT | IRa_SSC |
| IS_R | AAGCGATTCCGCAGATTGGTA |
| SI_F | AGAATCTTCGTAAACTGGGCT | SSC_IRb |
| SI_R | TCATTTCTTCTGGAACAATCACA |
| IL_F | TTCGTCGCCGTAAATAGGAA | IRb_LSC |
| IL_R | CTGGTTCACCGCTTTAGGTA |

**Supplementary Table S2.** PCR-based sequence validation of chloroplast junctions.

| Species | Location | PCR-based sequence (bp) | Start position | End position | Identities (%) | No. |
| --- | --- | --- | --- | --- | --- | --- |
| *I. ruthenica* | LSC_IRa | 1,006 | 82,215 | 83,220 | 100 |  |
|  | IRa_SSC | 954 | 107,698 | 108,651 | 100 |  |
|  | SSC_IRb | 514 | 126,298 | 126,811 | 100 |  |
|  | IRb_LSC | 872 | 151,691 | 287 | 100 |  |
| *I. uniflora* | LSC_IRa | 1,006 | 82,221 | 83,226 | 100 |  |
|  | IRa_SSC | 954 | 107,704 | 108,657 | 100 |  |
|  | SSC_IRb | 514 | 126,305 | 126,818 | 100 |  |
|  | IRb_LSC | 872 | 151,698 | 287 | 100 |  |

**Supplementary Table S3. Chloroplast genomes from NCBI used for phylogenetic analysis.**

| Family | No. | Taxon | GenBank accession number |
| --- | --- | --- | --- |
| Iridaceae | 1 | *Iris ruthenica* | OM037823 |
|  | 2 | *Iris uniflora* | OM037824 |
|  | 3 | *Iris sanguinea* | NC029227 |
|  | 4 | *Iris loczyi* | MT254070 |
|  | 5 | *Iris missouriensis* | NC042827 |
|  | 6 | *Iris gatesii* | NC024936 |
|  | 7 | *Iris tectorum* | MW201731 |
|  | 8 | *Iris domestica* | NC050833 |
|  | 9 | *Crocus cartwrightianus* | NC041459 |
|  | 10 | *Crocus sativus* | NC041460 |

Supplementary Table S4. Best-fitting substitution model selection using jModelTest for 72 protein-coding sequences.

|  | Model | | f(a) | f(c) | f(g) | | f(t) | | kappa | titv | Ra | | Rb | Rc | Rd | | Re | Rf | | pInv | gamma |
| --- | --- | --- | --- | --- | --- | --- | --- | --- | --- | --- | --- | --- | --- | --- | --- | --- | --- | --- | --- | --- | --- |
| AIC | GTR +G | | 0.31 | 0.17 | 0.20 | | 0.30 | | 5.86 | 2.77 | 1.61 | | 4.54 | 0.27 | 0.45 | | 4.57 | 1 | | 0.72 | 0.022 |
| Model | | -lnL* | | | | K | | AIC | | | | Delta | | | | Weight | | | CumWeight | | |
| GTR+G | | 128316.7 | | | | 23 | | 256679.4 | | | | 1.60348 | | | | 0.227059 | | | 0.733267 | | |
| TVM+I+G | | 128316.9 | | | | 23 | | 256679.8 | | | | 2.02286 | | | | 0.184107 | | | 0.917374 | | |
| GTR+I+G | | 128316.7 | | | | 24 | | 256681.5 | | | | 3.62524 | | | | 0.082626 | | | 1 | | |
| TPM1uf+G | | 128355.6 | | | | 20 | | 256751.3 | | | | 73.44592 | | | | 5.70E-17 | | | 1 | | |
| TIM1+G | | 128355.6 | | | | 21 | | 256753.2 | | | | 75.38108 | | | | 2.17E-17 | | | 1 | | |
| TPM1uf+I+G | | 128355.6 | | | | 21 | | 256753.3 | | | | 75.4653 | | | | 2.08E-17 | | | 1 | | |
| TIM1+I+G | | 128355.6 | | | | 22 | | 256755.2 | | | | 77.40096 | | | | 7.89E-18 | | | 1 | | |
| TPM3uf+G | | 128510.9 | | | | 20 | | 257061.8 | | | | 383.9244 | | | | 2.17E-84 | | | 1 | | |
| TIM3+G | | 128510.7 | | | | 21 | | 257063.3 | | | | 385.4932 | | | | 9.90E-85 | | | 1 | | |
| TPM3uf+I+G | | 128510.9 | | | | 21 | | 257063.8 | | | | 385.9465 | | | | 7.89E-85 | | | 1 | | |
| TIM3+I+G | | 128510.7 | | | | 22 | | 257065.3 | | | | 387.5159 | | | | 3.60E-85 | | | 1 | | |

Supplementary Table S5. Best-fitting substitution model selection using jModelTest for whole genome sequences.

|  | Model | | f(a) | f(c) | f(g) | | f(t) | | kappa | titv | Ra | | Rb | Rc | Rd | | Re | Rf | | pInv | gamma |
| --- | --- | --- | --- | --- | --- | --- | --- | --- | --- | --- | --- | --- | --- | --- | --- | --- | --- | --- | --- | --- | --- |
| AIC | GTR+I+G | | 0.30 | 0.19 | 0.19 | | 0.30 | | 3.33 | 1.58 | 0.9 | | 2.48 | 0.55 | 0.45 | | 2.40 | 1 | | 0.45 | 0.983 |
| Model | | -lnL* | | | | K | | AIC | | | | Delta | | | | Weight | | | CumWeight | | |
| GTR+I+G | | 290426.7 | | | | 28 | | 580909.3 | | | | 0 | | | | 0.540595 | | | 0.540595 | | |
| TVM+I+G | | 290427.8 | | | | 27 | | 580909.6 | | | | 0.32566 | | | | 0.459363 | | | 0.999958 | | |
| TIM1+I+G | | 290438.8 | | | | 26 | | 580929.6 | | | | 20.26112 | | | | 2.15E-05 | | | 0.999979 | | |
| TPM1uf+I+G | | 290439.8 | | | | 25 | | 580929.6 | | | | 20.33244 | | | | 2.08E-05 | | | 1 | | |
| GTR+G | | 290451.6 | | | | 27 | | 580957.3 | | | | 47.95432 | | | | 2.09E-11 | | | 1 | | |
| TVM+G | | 290452.9 | | | | 26 | | 580957.8 | | | | 48.50002 | | | | 1.59E-11 | | | 1 | | |
| TIM1+G | | 290464 | | | | 25 | | 580978 | | | | 68.69562 | | | | 6.54E-16 | | | 1 | | |
| TPM1uf+G | | 290465.1 | | | | 24 | | 580978.1 | | | | 68.8295 | | | | 6.12E-16 | | | 1 | | |
| GTR+I | | 290488.6 | | | | 27 | | 581031.2 | | | | 121.9116 | | | | 1.82E-27 | | | 1 | | |
| TVM+I | | 290489.9 | | | | 26 | | 581031.8 | | | | 122.5375 | | | | 1.33E-27 | | | 1 | | |
| TIM1+I | | 290501.3 | | | | 25 | | 581052.5 | | | | 143.2423 | | | | 4.25E-32 | | | 1 | | |

**Supplementary Table S6.** Raw and trimmed read data.

| Species | Input reads | Trimmed reads | | Total raw bases (bp) | Trimmed bases (bp) | |
| --- | --- | --- | --- | --- | --- | --- |
| *Iris uniflora* | 19,988,542 | 17,490,100 | 87.5% | 6,016,551,142 | 4,553,651,647 | 75.7% |
| *Iris ruthenica* | 17,454,052 | 15,573,854 | 89.2% | 5,253,669,652 | 2,880,177,520 | 54.8% |

Supplementary Table S7. Genome assembly information for *I. ruthenica* and *I. uniflora* chloroplast genomes.

| Species | Aligned reads (#) | Coverage (X) | Cp genome length (bp) |
| --- | --- | --- | --- |
| *I. ruthenica* | 423,465 | 739.3 | 152,275 |
| *I. uniflora* | 613,143 | 1,064.6 | 152,282 |

Supplementary Table S8. Genes in the chloroplast genomes of two *Iris* species.

| Group of genes | Name of genes |
| --- | --- |
| Photosystem I | *psaA*, *B*, *C*, *I*, *J*, *ycf3*^2^, *ycf4* |
| Photosystem II | *psbA*, *B*, *C*, *D*, *E*, *F*, *H*, *I*, *J*, *K*, *L*, *M*, *N*, *T*, *Z* |
| Cytochrome b6/f | *petA*, *B*^1^, *D*^1^, *G*, *L*, *N* |
| ATP synthase | *atpA*, *B*, *E*, *F*^1^, *H*, *I* |
| Rubisco | *rbcL* |
| NADH oxidoreductase | *ndhA*^1^, *B*^1,3^, *C*, *D*, *E*, *F*, *G*, *H*, *I*, *J*, *K* |
| Large subunit ribosomal proteins | *rpl2*^1,3^, *14*, *16*^1^, *20*, *22*, *23*^3^,*32*, *33*, *36* |
| Small subunit ribosomal proteins | *rps2*, *3*, *4*, *7*^3^, *8*, *11*, *12*^2,3,4^, *14*, *15*^3^, *16*^1^, *18*, *19* |
| RNA polymerase | *rpoA*, *B*, *C1*^1^, *C2* |
| Unknown function protein coding gene | *ycf1*^3^, *2*^3^ |
| Other genes | *accD*, *ccsA*, *cemA*, *clpP*^2^, *matK*, *infA* |
| Ribosomal RNAs | *rrn16*^3^, *23*^3^, *4.5*^3^, *5*^3^ |
| Transfer RNAs | *trnA-UGC*^1,3^*, trnC-GCA, trnD-GUC, trnE-UUC, trnF-GAA, trnfM-CAU, trnG-GCC, trnG-UCC, trnH-GUG, trnI-CAU*^3^*, trnI-GAU*^1,3^*, trnK-UUU, trnL-CAA*^3^*, trnI-GUA, trnL-UAA, trnL-UAG, trnM-CAU, trnN-GUU*^3^*, trnP-UGG, trnQ-UUG, trnR-ACG*^3^*, trnR-UCU, trnS-GCU, trnS-GGA, trnS-UGA, trnT-GGU, trnT-UGU, trnV-GAC*^3^*, trnV-UAC, trnW-CCA, trnY-GUA* |

^1^ Gene containing a single intron; ^2^ gene containing two introns; ^3^ two gene copies in IRs; ^4^ trans-splicing gene

Supplementary Table S9. Genic introns in *I. ruthenica* and *I. uniflora* chloroplast genomes.

| No. | Gene | Region | Exon I | Intron I | Exon II | Intron II | Exon III |
| --- | --- | --- | --- | --- | --- | --- | --- |
| 1 | *trnk-UUU* | LSC | 37 | 2622 | 35 |  |  |
| 2 | *rps16* | LSC | 36 | 826 | 216 |  |  |
| 3 | *trnG-UCC* | LSC | 23 | 675 | 48 |  |  |
| 4 | *atpF* | LSC | 144 | 779 | 411 |  |  |
| 5 | *rpoC1* | LSC | 432 | 725 (728) | 1617 |  |  |
| 6 | *ycf3* | LSC | 124 | 692 | 228 | 724 | 155 |
| 7 | *trnL-UAA* | LSC | 35 | 393 | 50 |  |  |
| 8 | *trnV-UAC* | LSC | 39 | 605 | 37 |  |  |
| 9 | *rps12* | LSC | 114 |  | 230 |  | 26 |
| 10 | *clpP* | LSC | 71 | 781 | 292 | 669 | 252 |
| 11 | *petB* | LSC | 8 | 746 | 646 |  |  |
| 12 | *petD* | LSC | 8 | 731 | 485 |  |  |
| 13 | *rpl16* | LSC | 9 | 886 | 403 |  |  |
| 14 | *rpl2* | LSC | 393 | 661 | 432 |  |  |
| 15 | *ndhB* | IR | 777 | 699 | 756 |  |  |
| 16 | *trnI-GAU* | IR | 42 | 940 (941) | 35 |  |  |
| 17 | *trnA-UGC* | IR | 38 | 809 | 35 |  |  |
| 18 | *ndhA* | SSC | 553 | 1059 | 539 |  |  |

^1^ Parentheses indicate *I. uniflora*


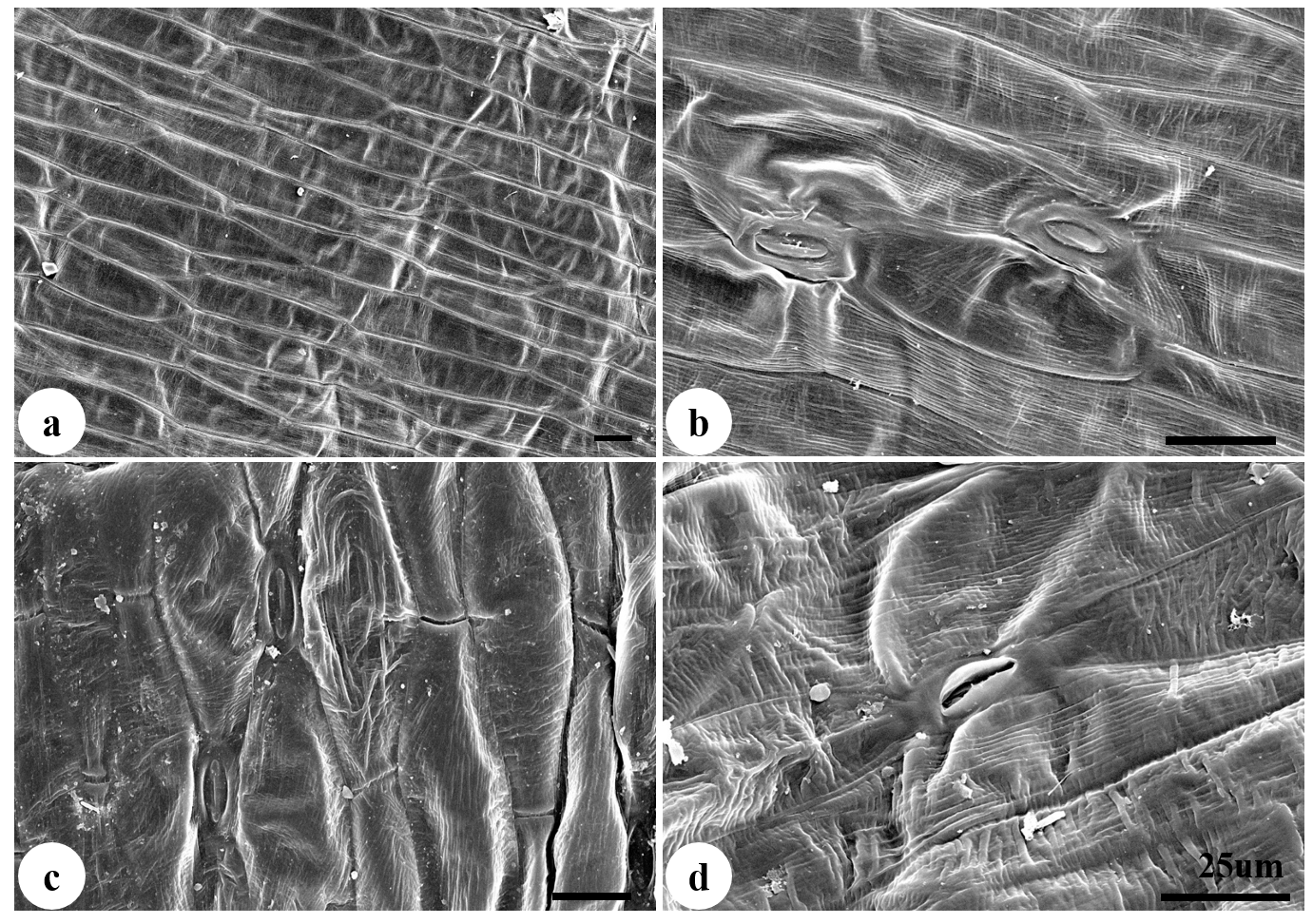


Supplementary Figure S1. Scanning electron micrographs of teapl surfaces in (a-b) *Iris ruthenica*, (c-d) *I. uniflora*. Scale bar = 25 μm

#
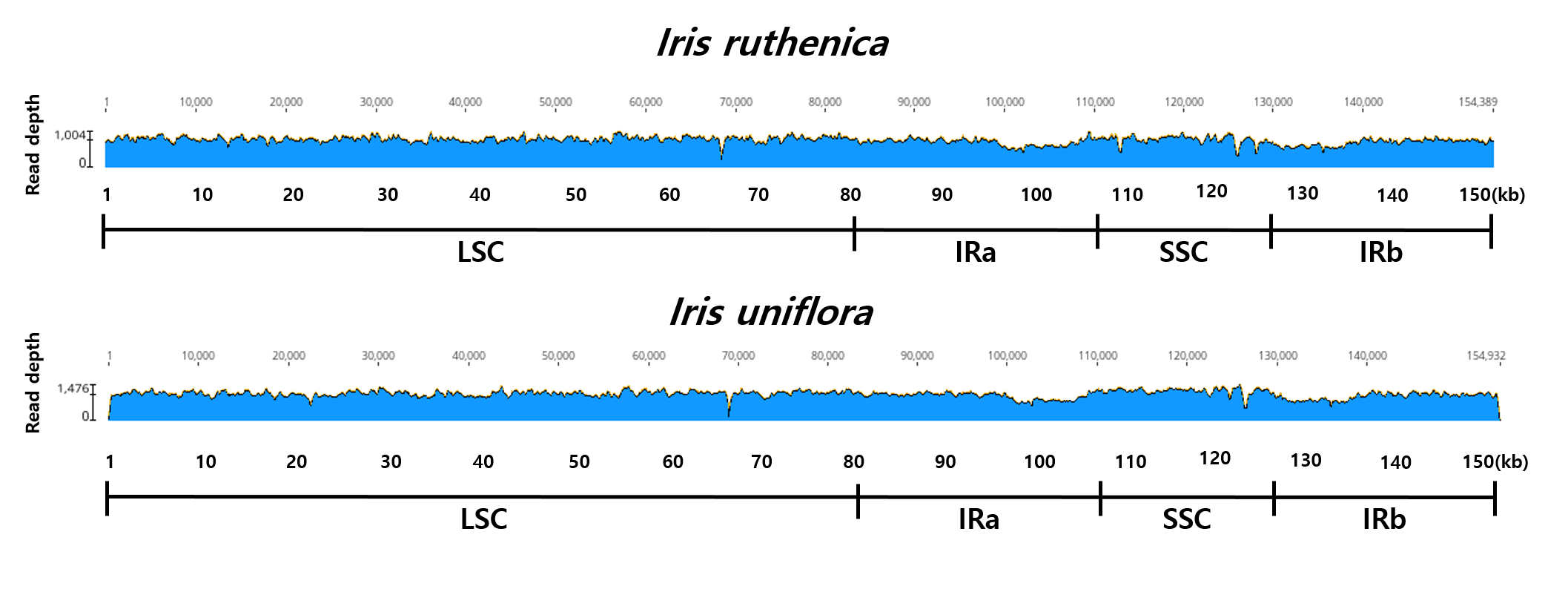
 Supplementary Figure S2. Distribution of paired-end reads mapped onto complete chloroplast genomes of *I. ruthenica* and *I. uniflora*. LSC, large single-copy region; SSC, small single-copy region; IRa, inverted repeat a; IRb, inverted repeat b.


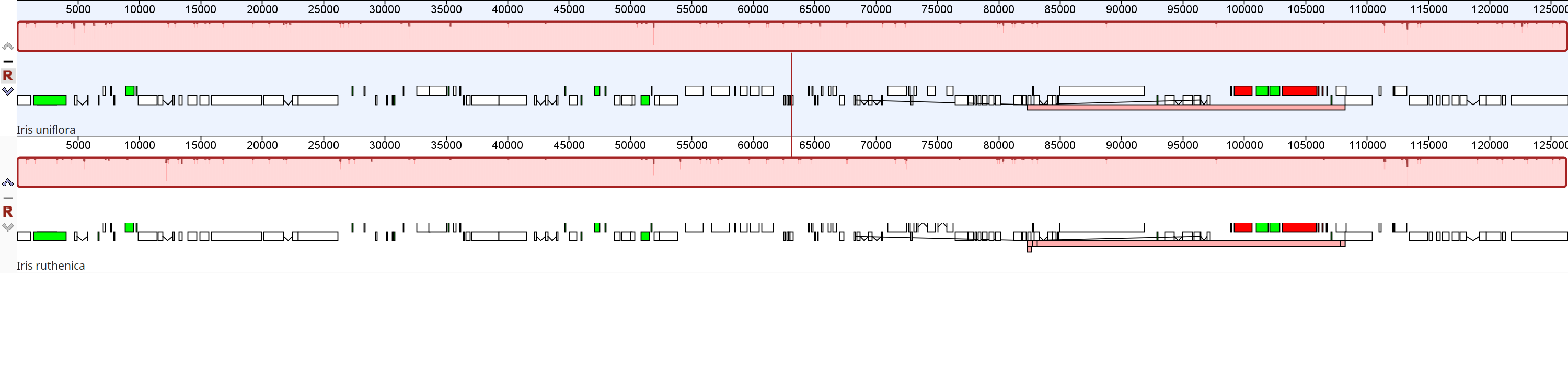


Supplementary Figure S3. Comparison of complete cp genomes from *I. ruthenica* and *I. uniflora* using the MAUVE algorithm.


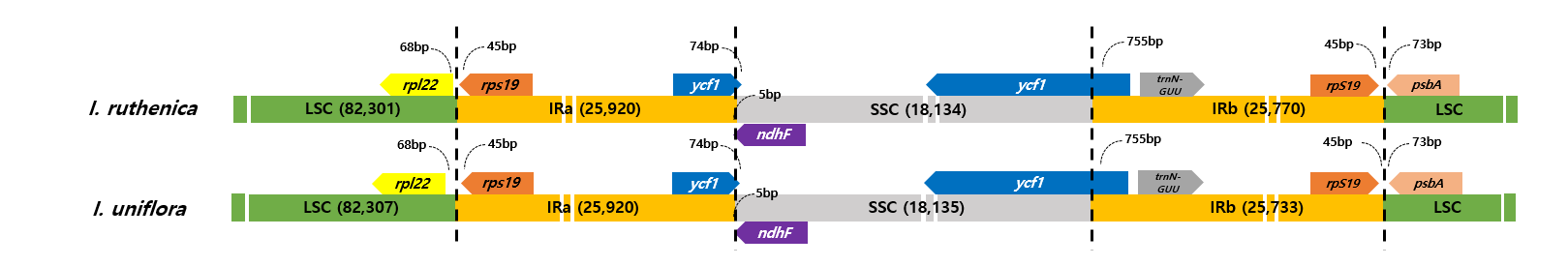


Supplementary Figure S4. Comparison of LSC, IR, and SSC junction positions among *I. ruthenica* and *I. uniflora* chloroplast genomes. LSC, large single-copy region; SSC, small single-copy region; IR, inverted repeat.


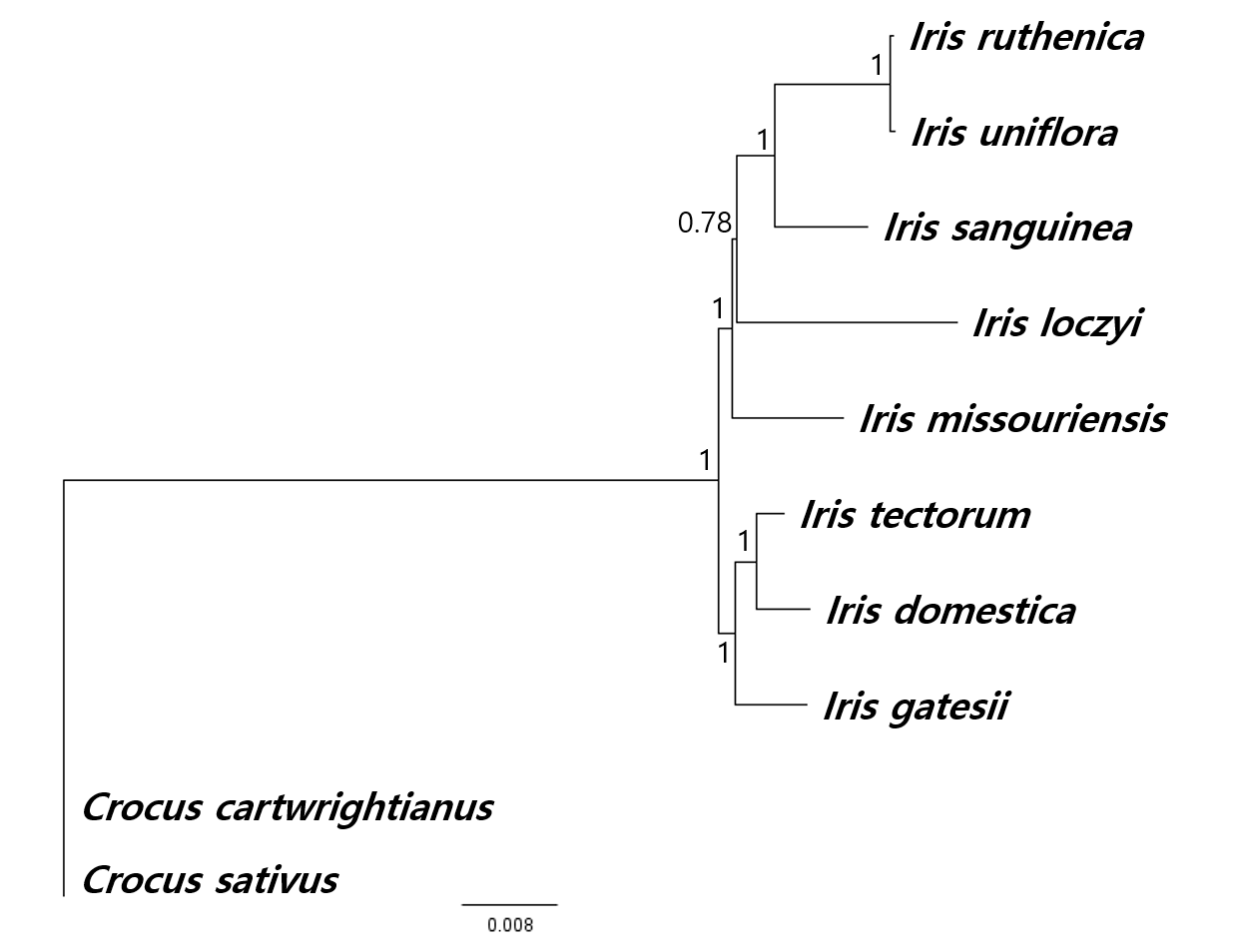


**Supplementary Figure S5.** Phylogenetic tree based on 74 protein-coding genes from eight *Irish* and two *Crocus* species using Bayesian posterior probabilities.

**Supplementary Figure S6.** Phylogenetic tree based on whole genome sequences from eight *Irish* and two *Crocus* species using maximum likelihood
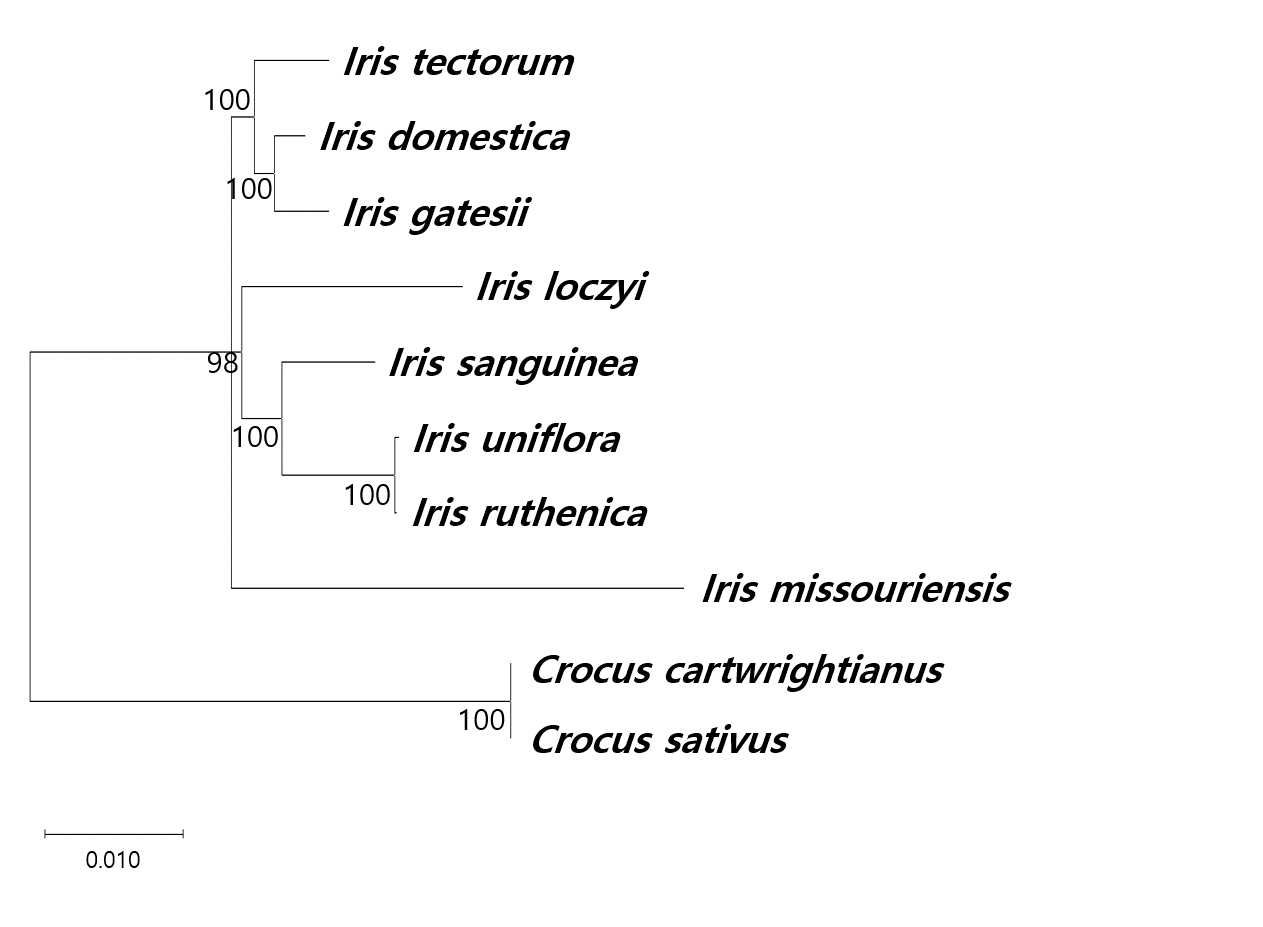
.


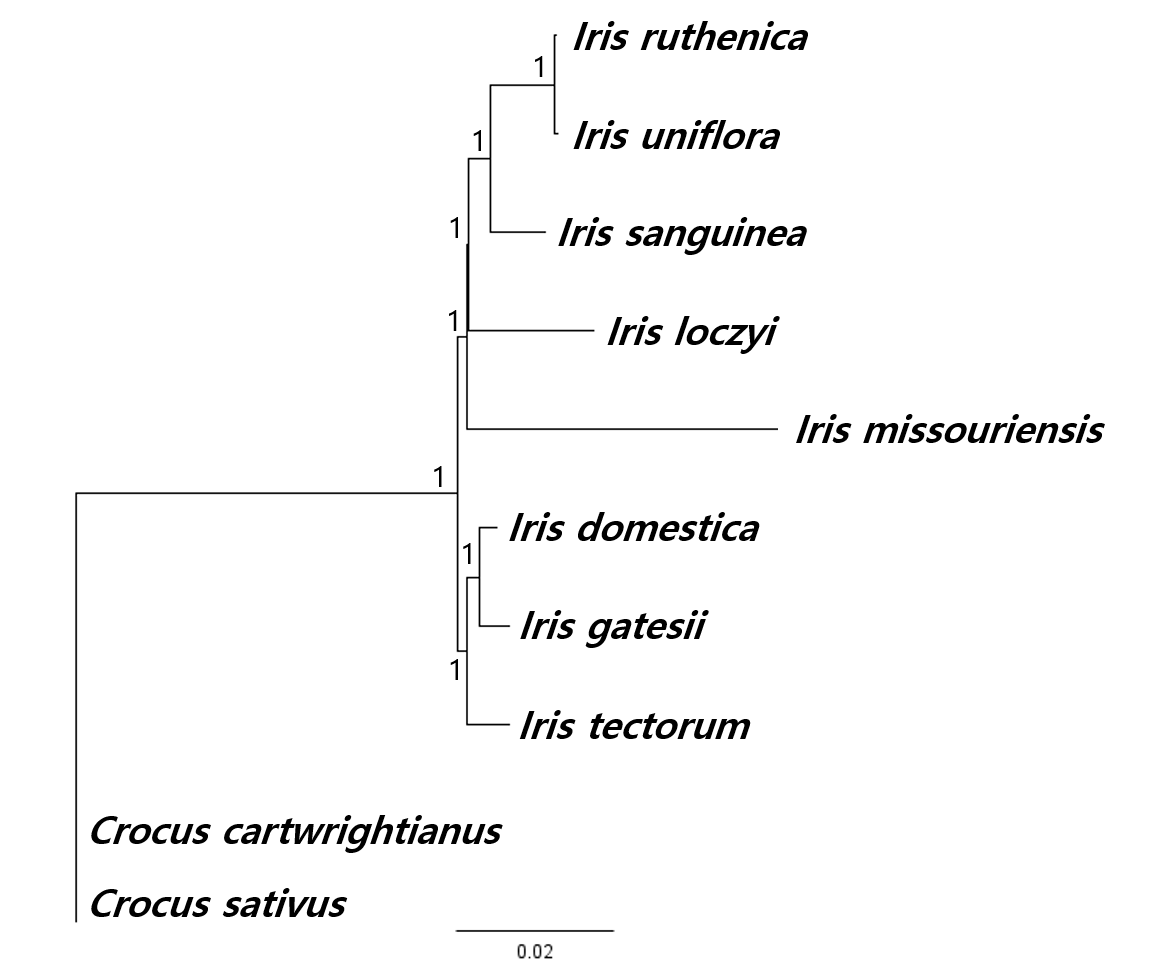
 **Supplementary Figure S7.** Phylogenetic tree based on whole genome sequences from eight *Irish* and two *Crocus* species using Bayesian posterior probabilities.
